# Comparative histopathology of virulent and avirulent *Meloidogyne javanica* populations on susceptible and resistant tomato plants

**DOI:** 10.3389/fpls.2024.1425336

**Published:** 2024-08-23

**Authors:** Márcia Gabriel, Marcilene F. A. Santos, Vanessa S. Mattos, Ana Cristina M. M. Gomes, Sheila F. de Almeida, Philippe Castagnone-Sereno, Leonardo S. Boiteux, Juvenil E. Cares, Regina M. D. G. Carneiro

**Affiliations:** ^1^ Universidade Federal de Santa Maria, Santa Maria–RS, Dep. de Agronomia, Brazil; ^2^ Embrapa Recursos Genéticos e Biotecnologia (Cenargen), Brasília, Brazil; ^3^ Universidade de Brasília, Dep. de Fitopatologia, Brasília, DF, Brazil; ^4^ INRAE, Université Côte d’Azur, CNRS, ISA, Sophia Antipolis, France; ^5^ Embrapa Hortaliças (CNPH), Brasília, Brazil

**Keywords:** *Solanum lycopersicum*, *S. peruvianum*, Mi-1.2 gene, hypersensitivity reaction. HR : realce, root-knot nematode

## Abstract

The *Mi-*1.2 gene confers resistance to a wide range of *Meloidogyne* species, being the most important resistance factor employed in tomato breeding so far. However, many aspects related to the interaction of *Mi-*1.2-carrying tomato cultivars and virulent/avirulent *Meloidogyne* populations have not yet been clarified. Herein, comparative histopathological analyses were carried after inoculation of the homozygous (*Mi*-1.2/*Mi*-1.2) tomato rootstock ‘Guardião’ and the susceptible cultivar ‘Santa Clara’ (*mi*-1.2/*mi*-1.2) with virulent and avirulent populations of *M. javanica.* In the susceptible control, it was possible to visualize second stage juveniles (J2) of avirulent population and feeding sites from 2 to 30 days after infection (DAI) with females reaching maturity at 24-34 DAI. In the resistant rootstock, the *Mi-*1.2 gene-mediated resistance was related mainly to early defense responses (pre-infection and hypersensitive reaction), which led to an immunity-like phenotype that completely prevented the reproduction of the avirulent *Meloidogyne* population. On the other hand, J2s of the virulent *M. javanica* population were able to penetrate roots much more than the avirulent population, migrated and developed normally, showing intense and similar pattern of penetration from 4 to 34 DAI in the root tissues of both resistant and susceptible tomato genotypes. The total numbers of J2, J3, J4, and females counted in ‘Santa Clara’ for the virulent population of *M. javanica* were higher than in ‘Guardião’.

## Introduction

The root-knot nematodes (RKN), genus *Meloidogyne* Göldi, 1887 have a cosmopolitan distribution, inducing severe damages in a wide range of economically important host plants (Maleita et al., 2011). In several crops, including Solanaceae species, the problems induced by *Meloidogyne* species can be controlled through resistance genes (Williamson and Kumar, 2006; Fuller et al., 2008). Resistance to RKNs in tomato is conferred by a single dominant gene, *Mi*-1.2, located on chromosome 6, which originated from the wild tomato species *Solanum peruvianum* L (Smith, 1944). The *Mi*-1.2 gene confers resistance against populations of 13 *Meloidogyne* species occurring in Brazil: *M. javanica* (Treub, 1885) Chitwood, 1949, *M. incognita* (Kofoid & White, 1919) Chitwood, 1949, *M. arenaria* (Neal, 1889) Chitwood, 1949, *M. morocciensis* Rammah & Hirschmann, 1990, *M. ethiopica *Whitehead, 1968, *M. inornata *Lordello, 1956, *M. luci* Maleita, Esteves, Cardoso, Cunha, Carneiro & Abrantes, 2018, *M. konaensis* Eisenback, Bernard & Schmitt, 1994, *M. paranaensis* Carneiro, Carneiro, Abrantes, Santos & Almeida, *M. izalcoensis* Carneiro, Almeida, Gomes & Hernandez, 2005, *M. petuniae* Charchar, Eisenback & Hirschmann, 1999 and *M. exigua* Göldi, 1887 (Gabriel et al., 2020).

RKNs induce substantial modifications of root ultrastructure and morphology, resulting in the formation of giant cells and galls (Huang and Maggenti, 1969). By blocking nematode development in the roots, the *Mi*-1.2 gene prevents these disturbances. However, in a few specific situations, the gene may lose its effectiveness. In particular, at continuous soil temperatures above 28°C (Holzmann, 1965), or when virulent populations are present (Castagnone-Sereno et al., 1994a; Roberts, 1995; Ornat et al., 2001; Devran and Söğüt, 2010). Furthermore, the heterozygous *versus* homozygous allelic state (allelic dosage) of the *Mi-*1.2 gene was shown to reduce the level of resistance expression (Jacquet et al., 2005; Maleita et al., 2011; Iberkleid et al., 2014; Gabriel et al., 2024). The mechanisms of plant resistance to nematodes can occur during pre-penetration or post-penetration. Pre-penetration resistance mechanisms prevent the invasion of plant roots by the nematodes. During this phase, the production of chemical substances in root exudates inhibits nematode attraction, or the reinforcement of physical barriers via the accumulation of cell wall–strengthening compounds such as lignin and callose (Mitsumasu et al., 2015) block nematode entry in root tissues. In the post-penetration resistance mechanisms, the invasion of J2s triggers a cascade of both local and systemic physiological and molecular processes in the host plant, including increased calcium flux, a burst of reactive oxygen species and defence gene expression (Williamson and Kumar, 2006; Sato et al., 2019; Rutter et al., 2022). These mechanisms act to impede or delay the migration or development of the nematode, thereby inhibiting the formation of feeding sites and/or limiting the reproduction of females.

The reproduction of *Meloidogyne* spp. virulent populations in resistant tomato cultivars bearing the *Mi-*1.2 gene has been reported across several countries, leading to a limitation of this strategy of control. Such nematode populations can be naturally virulent, that is, without the selection pressure exerted by previous exposure to a resistant cultivar (Ornat et al., 2001; Silva et al., 2019; Gabriel et al., 2022), or after repeated exposure to resistant cultivars in the field (Devran and Söğüt, 2010; Tzortzakakis et al., 2014; Gabriel et al., 2022) or under controlled conditions (Castagnone-Sereno et al., 1994b; Williamson, 1998).

Some resistance mechanisms have been observed in plants parasitized by RKNs, including the hypersensitive reaction (HR) due to the accumulation of phenolic compounds and the formation of phytoalexins and the activity of phenylalanine ammonia-lyase (PAL) related enzymes, *b*-glucanase, peroxidase, and polyphenol oxidase, among others (Sato et al., 2019). This is the case of tomato plants carrying the *Mi-*1.2 gene, where the primary resistance mechanism takes place in the first days after nematode infection, triggering the HR and working as a biochemical barrier blocking the development of second-stage juveniles (J2s) (Dropkin, 1969; Schaff et al., 2007). To the best of our knowledge, most of the information available on the histological features associated to the breaking of tomato resistance by RKN relate to the species *M. incognita.* The objective of the present study was to provide and analyse comparative data on nematode infection and the plant anatomical responses induced by avirulent and virulent populations using the tomato/*M. javanica* pathosystem. For that purpose, we developed two complementary experimental approaches: (*i*) the quantification of nematode penetration and development of avirulent and virulent *M. javanica* populations inoculated to susceptible and resistant tomato plants; (*ii*) the analysis of the histopathological changes associated with infection by avirulent and virulent populations of *M. javanica* into resistant and susceptible tomato cultivars.

## Materials and methods

### Nematode populations

Two populations of *M. javanica* sampled on tomatoes in Brazil were used in the study, one virulent to the *Mi*-1.2 gene from Frederico Westphalen, RS (27° 21’ 32’’ S/53° 23’ 38’’ O, cv.’Guardião’) and the other avirulent from Rodeio Bonito, RS (27° 28’ 15’’ S/53° 10’ 08’’ O, cv. Kada). They were previously identified by esterase phenotype Est J3 by Gabriel et al. (2022) and confirmed using the same enzymatic characterization, according to the methodology described by Carneiro and Almeida (2001).

### Plant material

The susceptible tomato (*Solanum lycopersicum* L.) cv. ‘Santa Clara’(*mi*-1.2/*mi*-1.2) and the resistant rootstock cv. ‘Guardião (*Mi*-1.2/*Mi*-1.2), homozygous for the *Mi-1.2* gene were studied previously (Gabriel et al., 2022) and used in the experiments. Seeds were purchased at Vegetal AgroNegócio in Brasília, DF.

### Inoculum preparation

The nematode populations were multiplied on cv. ‘Santa Clara’ and kept in a greenhouse under temperatures ranging from 23 °C to 28 °C. The suspension of eggs from each population was obtained according to the methodology of Hussey and Barker (1973) by grinding the roots in a blender with 0.5% sodium hypochlorite for approximately 30 seconds. The juveniles used as inocula in histopathological studies were obtained by hatching of nematode eggs in modified Baermann funnels collected for one week under 25° C (Flegg, 1967). The inoculum is counted using a Peter’s slide and calibrated with dilutions.

### Quantification of nematode penetration and development

Experiments were conducted in a greenhouse under temperatures ranging from 24 to 28°C in 2 L pots, using fine sand texture. The substrate was sterilized using autoclave at 120°C for 2 hours. To study the penetration and development of the two nematode populations inside the roots of the susceptible and resistant cv. ‘Santa Clara’ and ‘Guardião’, respectively. Tomato plants were cultivated in pots containing sterilized sand and fertilized with Forth Cote (15-09-12). Fifteen days after emergence, the seedlings were inoculated with the two populations separately. For this, 10,000 second-stage juveniles (J2s) were placed in four holes close to the stem of each plant, 2,500 in each hole. Plants of each cultivar (three replicates) were carefully removed from the pots at 2, 4, 7, 9, 11, 13, 16, 21, 27, and 34 days after inoculation (DAI). Their roots were carefully washed with tap water and stained with acid fuchsin, as described by Byrd et al. (1983). Subsequently, 40 stained root segments were cut under a stereomicroscope to observe and quantify the penetration and localization of J2s and to follow the subsequent development of the nematodes inside of the the roots (J3, J4, females and males). Root fragments showing infection by nematodes were mounted on slides for examination under an optical microscope and photographed (AxioPhot; Zeiss). The statistical analyses of the two experiment were performed using the SISVAR system, for each sample, the number of individuals was transformed to √(x+1) to normalize data and, after analysis of variance, the means were compared using Scott–Knott’s test at the 5% probability level (Scott and Knott, 1974).

### Histopathological studies

In parallel, three other root systems of each combination nematode population/tomato cultivar were sliced in thin sections of 2.5µm using a Leica ultracut UCT. Unstained root fragments, either showing galls or thickening or without symptoms were cut under a stereomicroscope. Approximately 60 root tips per DAI were analyzed at different times per treatment, and then fixed and embedded in Technovit 7100 epoxy resin (Kulzer Friedrichsdorf), as described by Pegard et al. (2005), and following manufacturer’s recommendations. Unstained root sections were mounted on glass slides, and fluorescence was observed under ultraviolet (UV) light, using a filter of 365-395 nm. The same sections were stained (1 min. at 60°C) with 0.5% toluidine blue in 0.1 M sodium phosphate buffer, pH 5.5, and observed using alight microscopy (AxioPhot, Zeiss). More than 5,000 sections of each treatment were visualized and documented.

## Results

### Avirulent *Meloidogyne javanica*

Inoculation with the avirulent *M. javanica* population confirmed the host status of both tomato genotypes: nematodes could massively invade and develop in the roots of the susceptible cv. ‘Santa Clara’, while their penetration and further development were almost totally impaired in the roots of the resistant cv. ‘Guardião’ (*P* ≤ 0.05; **Table 1**). Very few J2 were observed in the roots of the resistant cultivar (two and three at 4 and 7 DAI, respectively), and no other developmental stages could be detected, excepted three females without egg-masses at 34 DAI (**Table 1**). In the susceptible cultivar, the major steps of nematode development kinetics were observed as follows: J2 from 4 to 16 DAI; J3 from 11 to 34 DAI; J4 from 13 to 34 DAI; females without egg-masses from 24 to 34 DAI and females starting to lay eggs at 34 DAI, respectively (**Table 1**). No males were observed in the roots of either the susceptible or the resistant tomatoes ‘Santa Clara’ and ‘Guardião’, respectively.

**Table 1:** Number of second-stage juveniles (J2) penetration and development of third (J3) and fourth (J4) nematode stages, of avirulent *Meloidogyne javanica* population in 40 sections of three tomato roots repetitions of the cultivar ‘Santa Clara’ and rootstock ‘Guardião’ inoculated with 10,000 J2.

		4° DAI*	7° DAI	9° DAI	11° DAI	13° DAI	16° DAI	24° DAI	34° DAI	Nematodes **
J2	Guardião	2	3	0	0	0	0	0	0	5 b
	S. Clara	96	259	274	123	55	31	1	1	840 a
J3	Guardião	0	0	0	0	0	0	0	0	0 b
	S. Clara	0	0	0	229	192	56	50	16	543 a
J4	Guardião	0	0	0	0	0	0	0	0	0 b
	S. Clara	0	0	0	6	154	383	147	156	846 a
Females without egg mass	Guardião S. Clara	0 0	0 0	0 0	0 0	0 0	0 0	3 498	0 445	3 b 943 a
Females laying eggs	Guardião	0	0	0	0	0	0	0	0	0 b
	S. Clara	0	0	0	0	0	0	0	63	63 a
Male	Guardião	0	0	0	0	0	0	0	0	0
	S. Clara	0	0	0	0	0	0	0	0	0

* DAI: Days after J2 inoculation; ** Means of total nematodes followed by the same lowercase letter for different RKN stages in the column do not differ statistically from each other according to the Scott-Knott test at 5% probability (P ≤ 0.05). Cv = 28.1 (virulent population) and Cv = 26.4 (avirulent population).

**Figure 1:**
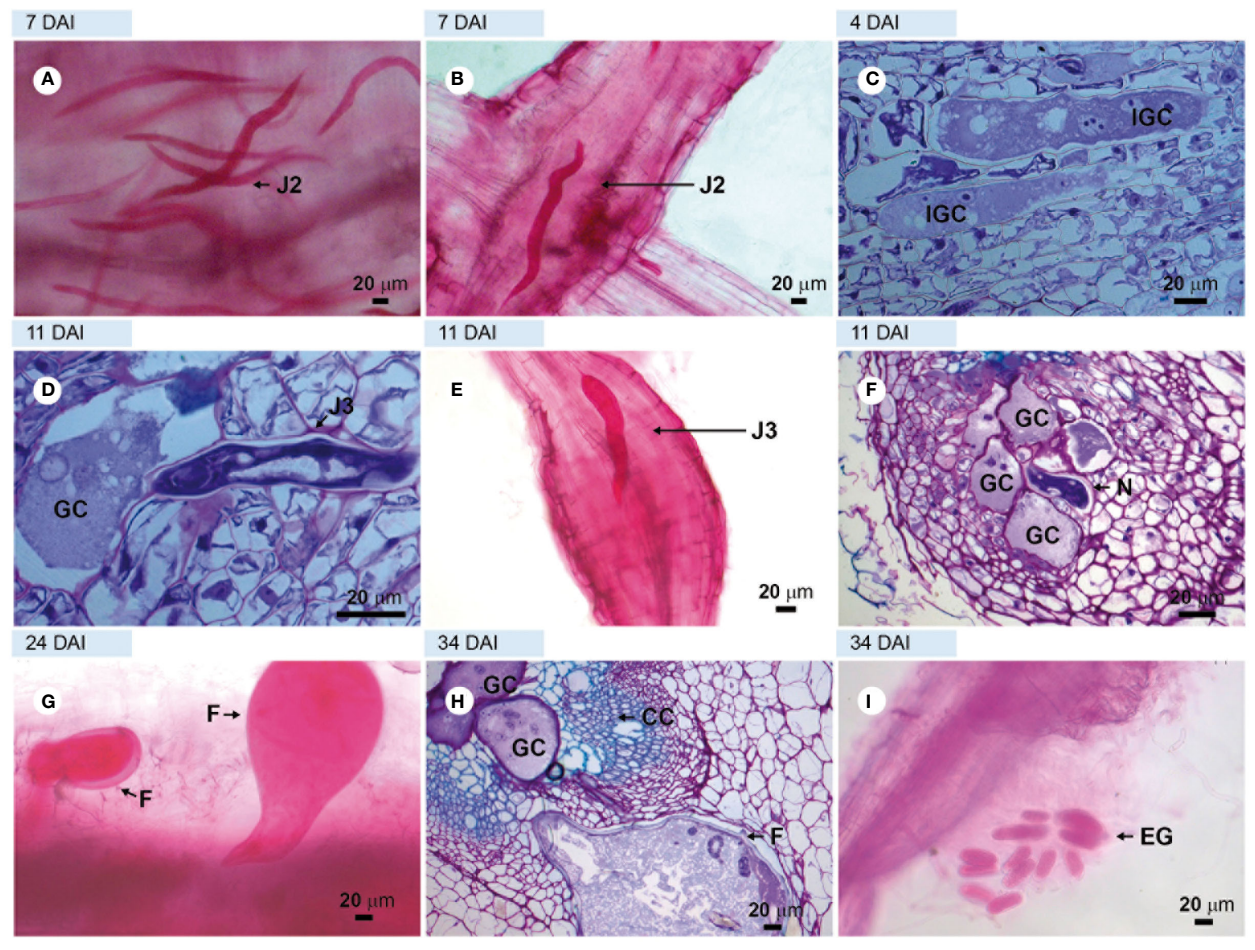
Compatible interaction observed in the susceptible tomato plant. Roots of cv. Santa Clara (control) infected with avirulent *Meloidogyne javanica*. **(A, B, E, G, I)** in light microscopy (LM) observations of root fragments stained with acid fuchsin. Sections **(C, D, F)**; and H = roots stained with toluidine blue. **(A)** = second-stage juveniles (J2s) in the cortex and vascular cylinder, and **(B)** = swollen J2s into vascular cylinder. **(C)** = sections showing the beginning of giant cell formation at 4 DAI; **(D)** = third-stage juvenile (J3) next to giant cells; **(E)** = third-stage juvenile (J3) in the vascular cylinder and root enlargement; **(F)** = giant cells formed next to J4; **(G)** = young females; **(H)** = adult female and giant cells with multiple nucleus and thick cell wall and **(I)** = egg mass at 34 DAI. DAI, days after inoculation; N, nematode; GC, giant cell; IGC, initial giant cell; EG, egg mass; YF, young female; CC, vascular cylinder.

Observation of the roots of the susceptible cv. ‘Santa Clara’ stained with acid fuchsin at 4–16 DAI revealed the presence of numerous J2 (**Figure 1A**) in the cortical region with some of them in the vascular cylinder (a location compatible with the initiation of feeding sites) (**Figures 1A, B**). Light microscopic observations of root sections stained with toluidine blue at 4 DAI confirmed this time point as the beginning of giant cell formation in the vascular tissue, as observed in longitudinal sections (**Figure 1C**). At 11 DAI, giant cells were observed next to third-stage juveniles (J3; **Figure 1D**), along with root enlargement (**Figure 1E**), and next to fourth-stage juveniles (J4), with cell wall thickening noticed between giant cells (**Figure 1F**). At 24 DAI, young females were clearly visible in acid fuchsin stained tissues (**Figure 1G**) and at 34 DAI, adult females were observed next to multinucleate giant cells in the vascular cylinder, together with egg-masses extruded outside the root (**Figures 1H, I** respectively), thus completing the nematode life cycle.

**Figure 2:**
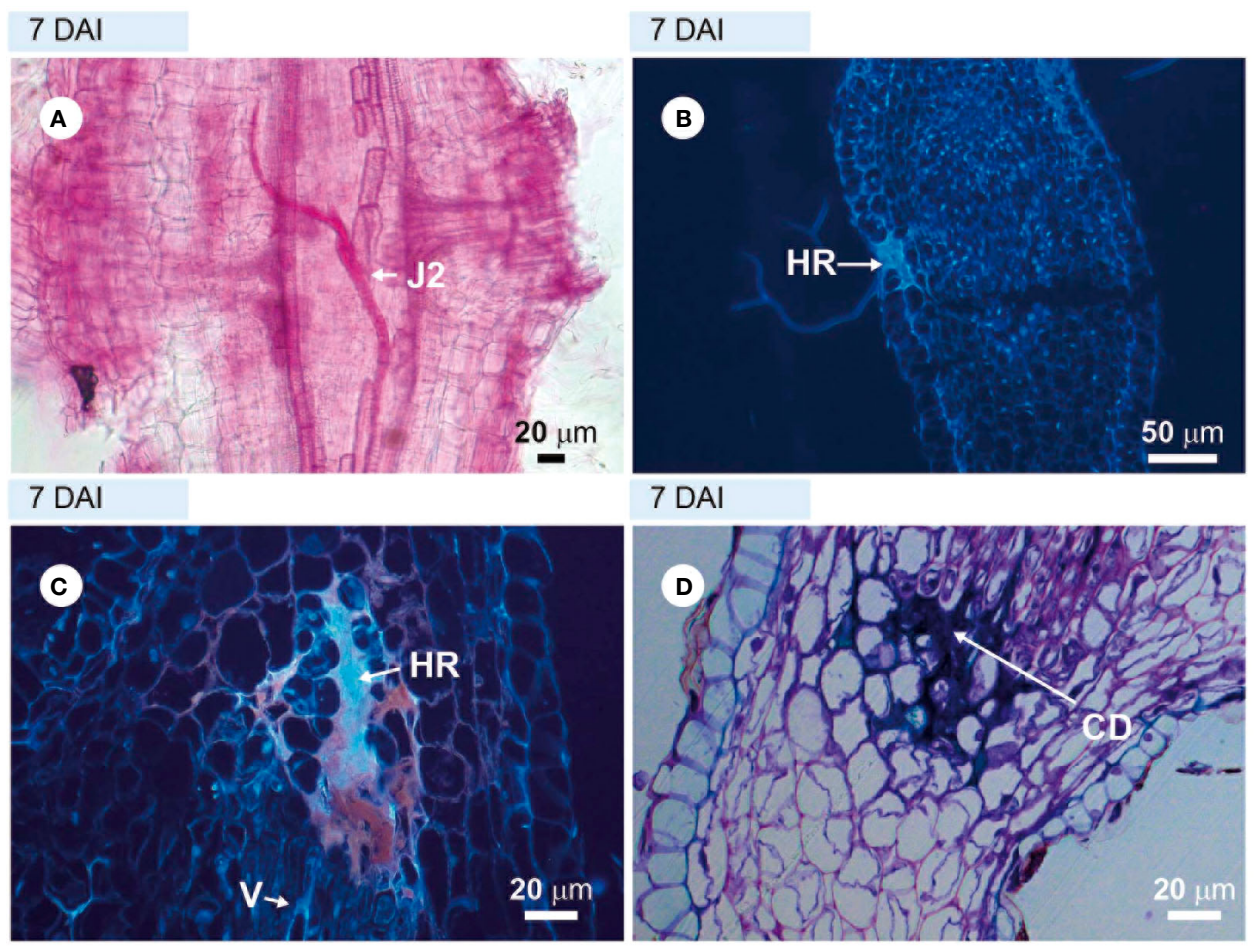
Incompatible interaction observed in roots of the resistant hybrid tomato plant ‘Guardião’ infected with avirulent *Meloidogyne javanica*. **(A)** = light microscopy observations of root fragment stained with acid fuchsin. **(B, C)** = unstained sections visualized under UV light; **(D)** = section stained with toluidine blue, showing cell death (CD). **(A)** = second-stage juvenile (J2) inside the root; **(B)** and **(C)** = hypersensitivity reaction (HR). DAI, days after inoculation; V, vessel.

The roots of the highly resistant rootstock cv. ‘Guardião’, stained with acid fuchsin, showed that very few avirulent *M. javanica* J2 could penetrate the roots at 4–7 DAI (**Figure 2A**). Through sections visualized under ultraviolet light (UV), obtained at 7 DAI, it was possible to observe a hypersensitive reaction (HR) at several locations in the cortical region: epidermis, parenchymatous cortex (**Figures 2B, C**) close to the vascular cylinder. When stained with toluidine blue, these sections appeared strongly stained at the same sites (**Figure 2D**), a signal characteristic of cell death. Despite numerous observations from 4 to 34 DAI, it was not possible to detect any other nematode stages within the vascular cylinder cells in the root tissues of the resistant rootstock, suggesting an early resistance mechanism closely related to immunity.

### Virulent *Meloidogyne javanica*

As expected, the *M. javanica* virulent population was able to infect, develop and reproduce on the resistant rootstock cv. ‘Guardião’, as well as on the susceptible cv. ‘Santa Clara’. After staining the roots with acid fuchsin, monitoring of inoculations revealed identical development kinetics on both cultivars (*P* ≤ 0.05; **Table 2**), and very similar to that previously observed for the avirulent population on the susceptible cultivar (**Table 1**). In terms of nematodes counted in the roots, significantly higher cumulative numbers of individuals were found in the susceptible cv. ‘Santa Clara’ compared with the resistant cv. ‘Guardião’ for the J2, J3 and female with egg mass stages, and equivalent numbers for the other developmental stages (*P* ≤ 0.05; **Table 2**). Some males were observed on both cv. from 21 to 27 DAI.

**Table 2:** Number of second-stage juveniles (J2) penetration and development of third (J3) and fourth (J4) nematode stages, of virulent *Meloidogyne javanica* population in 40 sections of three tomato roots repetitions of the cultivar ‘Santa Clara’ and rootstock ‘Guardião’ inoculated with 10,000 J2.

		2°	4°	7°	9°	11°	13°	16°	21°	27°	34°	Nematodes
		DAI	DAI	DAI	DAI	DAI	DAI	DAI	DAI	DAI	DAI	**
J2	Guardião	2	253	444	322	134	65	21	1	3	10	1255 b
	S. Clara	4	374	540	424	236	110	6	3	8	34	1739 a
J3	Guardião	0	0	0	8	410	379	258	116	21	10	1202 b
	S. Clara	0	0	0	4	463	417	335	149	26	21	1415 a
J4	Guardião	0	0	0	0	4	199	389	187	116	14	909 a
	S. Clara	0	0	0	0	2	230	434	286	52	10	1014 a
Females without egg mass	Guardião	0	0	0	0	0	2	205	317	428	116	1068 a
	S. Clara	0	0	0	0	0	0	305	382	365	56	1108 a
Females laying eggs	Guardião	0	0	0	0	0	0	0	0	481	576	1057 b
	S. Clara	0	0	0	0	0	0	0	0	608	830	1438 a
Male	Guardião	0	0	0	0	0	0	0	0	9	64	73
	S. Clara	0	0	0	0	0	0	0	9	14	8	31

*DAI, Days after J2 inoculation; ** Means of total nematodes followed by the same lowercase letter for different RKN stages in the column do not differ statistically from each other according to the Scott-Knott test at 5% probability (P ≤ 0.05). Cv = 28.1 (virulent population) and Cv = 26.4 (avirulent population).

**Figure 3:**
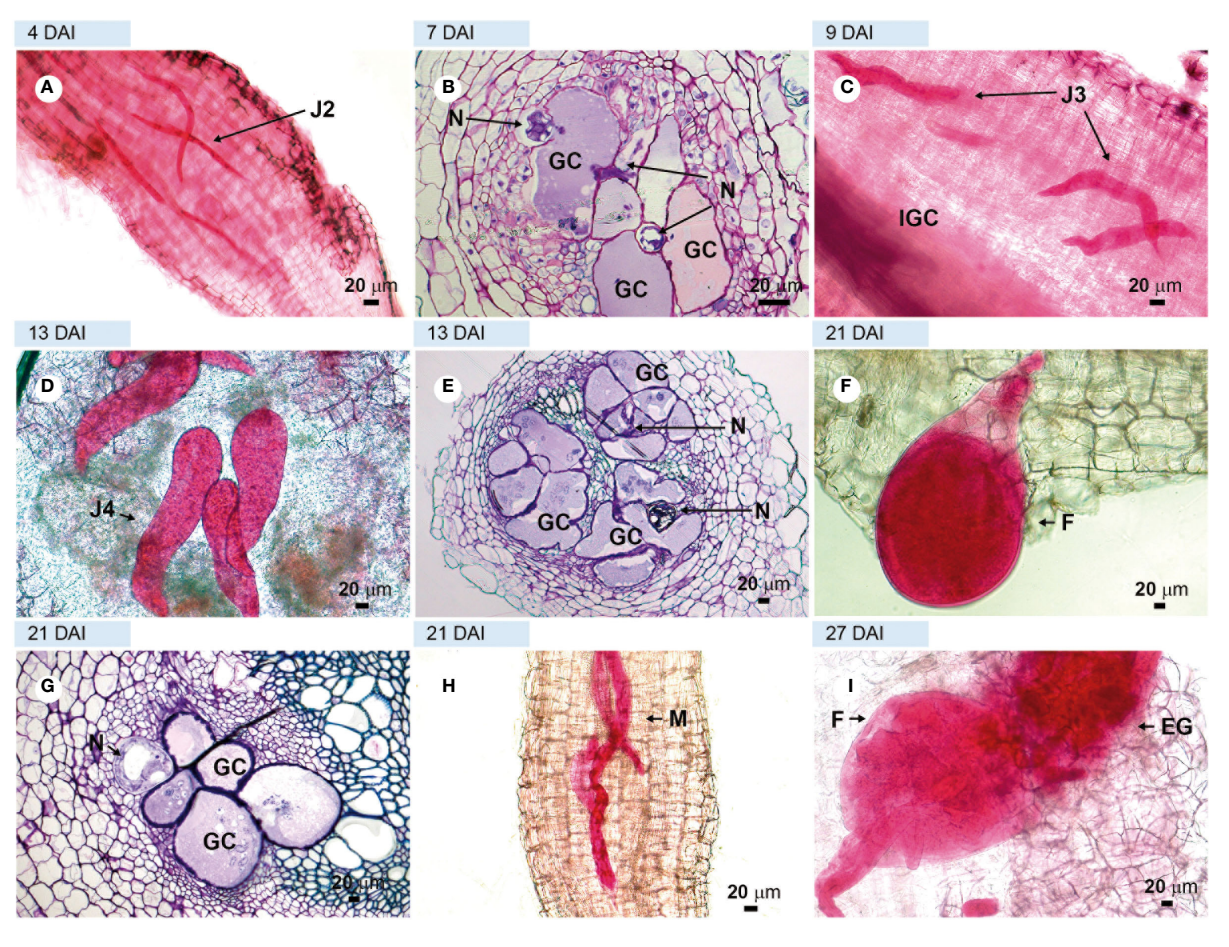
Compatible interaction. Roots of tomato plant cv. ‘Santa Clara’ susceptible, infected with virulent *Meloidogyne javanica*. **(A, C, D, F, H, I)** = light microscopy observations of root fragments stained with acid fuchsin. **(B, E, G)** = sections stained with toluidine blue. **(A)** = second stage juveniles (J2) inside the roots at 4 DAI; **(B)** = initial giant cell formation in the vascular cylinder; **C** = third-stage juvenile (J3); **(D)** = fourth-stage juvenile (J4) in the vascular cylinder. **(E)** = large number of giant cells in the vascular cylinder; **(F)** = young female feeding in the vascular cylinder in a well-thickened root; **(G)** = well-formed giant cells; **(H)** = males in large numbers and **(I)** = adult female with egg-mass. DAI, days after inoculation; GC, giant cell; M, male; F, female; CC, vascular cylinder; N, nematodes.

**Figure 4:**
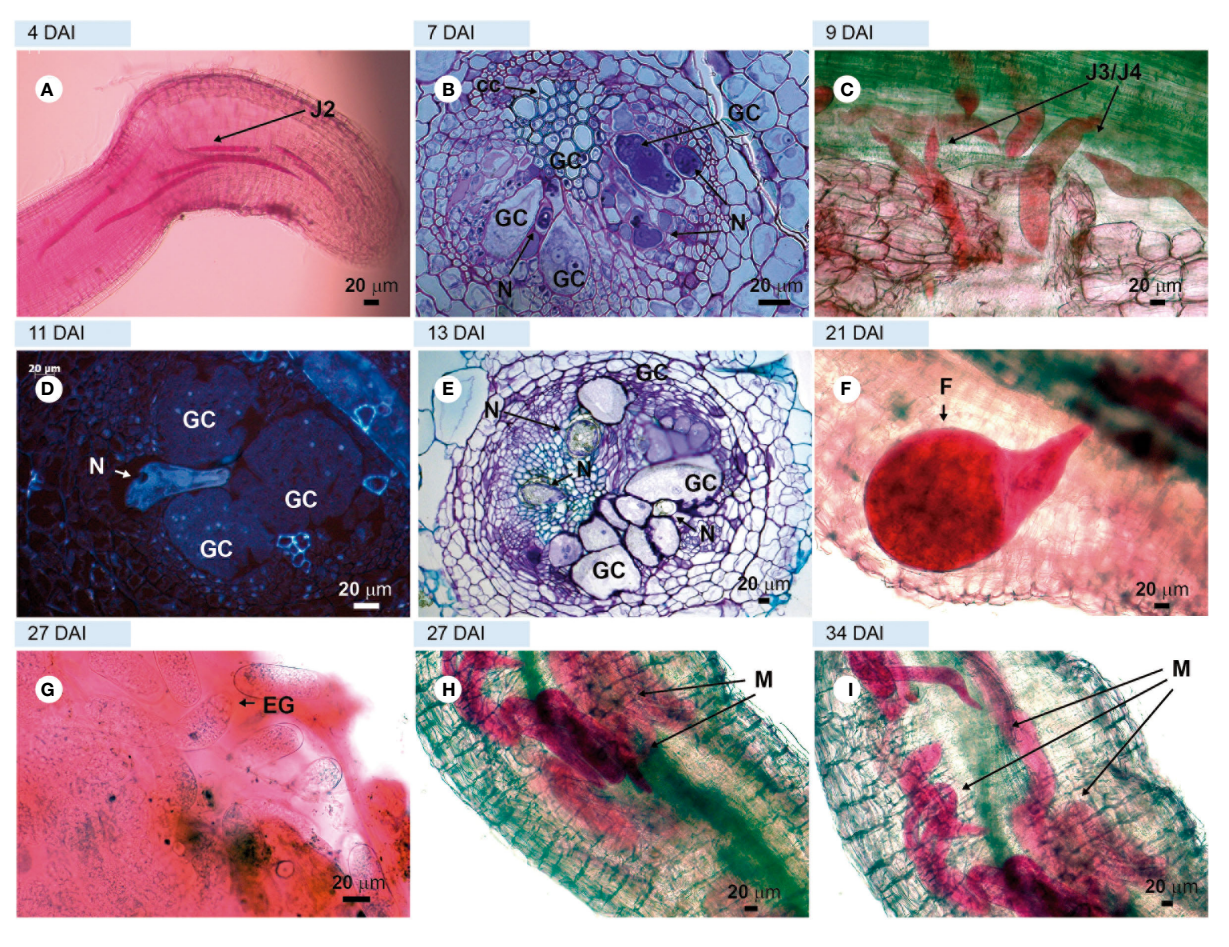
Compatible interaction. Tomato roots of ‘Guardian’ rootstock infected with virulent *Meloidogyne javanica*. **(A, C, F, G, H, I)** = light microscopy observations of a root fragment stained with acid fuchsin. **(B, E)** = sections stained with toluidine blue. D = unstained sections viewed under UV; **(A)** = second-stage juvenile (J2) in the apical region of the root; **(B)** = giant cell in formation; **(C)** = third and fourth-stage juveniles **(J3/J4)** in the vascular cylinder; **(D)** = well-formed feeding site without the presence of HR; **(E)** = thick-walled giant cells; **(F)** = female feeding into the vascular cylinder; **(G)** = eggs; **(H)** = male emerging from the J4 cuticle layers and **(I)** = a large number of males in different regions of the root: cortex and vascular cylinder. GC, giant cell; F, female; EG, eggs inside egg mass; HR, hypersensitivity reaction; N, nematodes.

Examinations of roots and root sections of the susceptible cv. ‘Santa Clara’ and the resistant cv. ‘Guardião’ stained with acid fuchsin showed that the virulent nematode population was equally able to penetrate roots and complete its development cycle up to the production of egg-masses by adult females on both cultivars. (**Figures 3A, C, D, F, H, I** and **Figures 4A, C, F, G**, respectively). The only notable difference was the presence of numerous males on ‘Guardião’ at 27 DAI. Males were observed releasing themselves from J4 cuticles inside the roots at 34 DAI (**Figures 4H, I**).

Histological observations revealed numerous feeding sites in both the susceptible and the resistant cultivars, with the formation of giant cells initiated in the vascular cylinder region at 7 DAI (**Figures 3B**, **4B**) and illustrated further at 13 and 27 DAI in roots of ‘Santa Clara’ (**Figures 3E–G**) and at 11 and 13 DAI in ‘Guardião’ (**Figures 4D, E**). The histological changes observed in the root cells of both the resistant and susceptible cultivars when parasitized by the virulent population of *M. javanica* exhibited a high degree of similarity. Additionally, these changes closely resembled those observed in roots of the susceptible cv. parasitized by the avirulent population of *M. javanica*.

## Discussion

Plant resistance responses to RKN are diverse and can occur either before or after pre-parasitic juveniles have penetrated root tissues (Rutter et al., 2022). The structure of the root itself can act as a physical barrier to J2 penetration, as has been observed in many plant species, e.g., in pepper (Pegard et al., 2005) or rice (Cabasan et al., 2012). However, it is often difficult to separate the early effects of these physical barriers to nematode penetration from the chemical defense responses that the host will initiate following RKN infection, thus leading to plant immunity. In the case of basal immunity, the plant recognizes nematode-derived molecules referred to as pathogen-associated molecular patterns (PAMPs) and activates a series of both local and systemic defense responses that include callose deposition, a burst of reactive oxygen species (ROS), and activation of defense gene expression, all these mechanisms being collectively known as PAMP - detected immunity (PTI) (Goode and Mitchum, 2022; Siddique et al., 2022). When the plant harbours dominant resistance (R) gene(s), nematode effectors are triggered by intracellular receptors, which in turn initiates programmed cell death within the host, a pathway designated as effector-triggered immunity (ETI). In particular, nucleotide-binding and leucine-rich repeat (NB-LRR) genes constitute most of known ETI R genes against RKN in dicotyledones, in both annual and perenial crops, e.g., *Mi-1.2* in tomato (Milligan et al., 1998) or *Ma* in plum (Claverie et al., 2011).

Previous histopathological studies of tomato plants harboring the *Mi*-*1.2* resistance gene have indicated that it mediates defense responses to *M. incognita* associated with the induction of an hypersensitive reaction (HR), which prevents the development of giant cells by blocking the parasite’s penetration in the root and the completion of its life cycle (Melillo et al., 2006). Here, our results showed that two different mechanisms might be involved in the expression of resistance of the tomato rootstock ‘Guardião’ and an avirulent *M. javanica* population. The first mechanism occurred as a strong pre-infection defense response that prevented the nematodes from penetrating the root epidermis, suggesting a potential association with physical or biochemical barriers. Although we did not observe any anatomical reinforcement of the root epidermis in the resistant plants, additional experiments are needed to specify more precisely which of these two mechanisms acts as a barrier to J2 penetration, or whether they act in combination. This early defense layer proved to be powerful and blocked about 99% of the J2 penetration compared to susceptible plants. Such barriers were previously suggested to occur in various crops, e.g., grape (Anwar and McKenry, 2000) or cotton (Anwar et al., 1994) One of the resistance mechanisms of tomato related to *Mi*-1.2 gene to RKN is inhibiting the penetration of juvenile (J2) during invasion. However, there is variation in numbers of penetrating J2, depending on the nematode population and tomato genotype but the reason is not clear (Wubie and Temesgen, 2019).

Complementary, a second resistance mechanism occurred as a post-infection defense response at 4-7 DAI, soon after J2 penetration in the root tissues, with HR-cell death observed in the cortex and vascular cylinder regions of the root, as has been observed in some tomato genotypes resistant to RKN (Dropkin, 1969; Regaieg and Horrigue-Raouani, 2012). This mechanism was described in several plant species hosts for other RKN species, including pepper (Pegard et al., 2005), cotton (Mota et al., 2012; Lopes et al., 2020), coffee (Lima et al., 2015) and rice (Mattos et al., 2019). Overall, very few J2s were able to penetrate the tomato roots, and were further blocked due to the HR. But this phenomenon was much less intense than the non-penetration of J2s into the roots, which may result from various, non-exclusive pathways: either the roots did not attract or even repelled J2s, or J2 penetrated then rapidly left the roots, although we could not get strong anatomical evidence for the latter option. For example, such protection was shown for *Cucumis sativus* L., in which the triterpene cucurbitacin isolated from root exudates repelled J2s (Hayne and Jones, 1976). Similarly, amino acids exuded from *Sesamum indicum* L. roots have a nematostatic effect on *Meloidogyne* J2 (Tanda et al., 1989). In the past decades, much emphasis has been given to the mechanisms linked to HR, but studies related to physical and chemical defense layers have been rather neglected, probably due to the technical difficulties in identifying plant molecules that modulate nematode behavior in soil (Siddique et al., 2022).

As expected, the penetration experiment and the histopathological observations of tomato roots inoculated with the *M. javanica* virulent population agree with the *in vivo* infection test we previously conducted with the same plant genotypes (Gabriel et al., 2024). The nematode established feeding sites and maintained healthy giant cells, containing several nuclei and thickened cell walls in roots of resistant tomato plants, similar to those observed in the susceptible cultivar ‘Santa Clara’. The ability of virulent RKN to induce feeding sites and complete their life cycle has been reported previously on tomato cultivars harbouring the *Mi-1.2* resistance gene (e.g., Regaieg and Horrigue-Raouani, 2012; Iberkleid et al., 2014; Ploeg et al., 2023). Here, we showed that the same observation can be done on a resistant rootstock. Variability in the level of reproduction of virulent RKN on resistant tomato has been documented, and possibly correlated to a dosage effect of the *Mi-1.2* gene when it is present in hetezygous allelic state (Tzortzakakis et al., 1998; Jacquet et al., 2005; Iberkleid et al., 2014). Better results were observed in our previous study where heterozygous tomato rootstocks were classified solely as resistant rather than as highly resistant in homozygous plants (Gabriel et al., 2024).

In summary, our findings indicate that the resistance to *M. javanica* conferred by the tomato *Mi-1.2* gene results in early plant responses involving both pre-infection mechanisms and HR, which ultimately prevent the nematode from completing its development cycle. In addition, we also demonstrated that nematodes virulent to this resistance gene are able to develop normally on both susceptible and resistant plants, and induce in both cases feeding sites identical to those observed in a compatible interaction between a susceptible tomato and an avirulent nematode. To our knowledge, this is the first report of an in-depth histological characterization of a *M. javanica* population able to overcome the resistance conferred by the tomato *Mi-1.2* gene. The broad implication of the present research is that virulent *M. javanica* populations may represent a major agronomic risk if widely dispersed in tomato crops. Since the long-term use of both homozygous and heterozygous resistant crop genotypes can lead to the emergence of virulent nematode populations, management of resistance genes in the field is of utmost importance to promote their durability. In particular, pyramiding of two different resistance genes in one genotype or alternating different resistance genes in rotation are strategies that suppressed or reduced the emergence of virulent RKN isolates, as demonstrated in sweet pepper (Djian-Caporalino et al., 2014). To that respect, combining the use *Mi-1.2* with other natural RKN resistance genes that have been identified in tomato (El-Sappah et al., 2019) is clearly a challenge for the future. In complement, the association of host resistance to other management practices, such as solarization, organic matter, wet fallow, and biological control, among others, will undoubtedly increase the control of RKN and consequently the long-term sustainability of tomato production.

## Data Availability

The original contributions presented in the study are included in the article/supplementary material. Further inquiries can be directed to the corresponding authors.
